# Ploidy Variation and Spontaneous Haploid-Diploid Switching of Candida glabrata Clinical Isolates

**DOI:** 10.1128/msphere.00260-22

**Published:** 2022-06-21

**Authors:** Qiushi Zheng, Jing Liu, Juanxiu Qin, Bingjie Wang, Jian Bing, Han Du, Min Li, Fangyou Yu, Guanghua Huang

**Affiliations:** a Shanghai Institute of Infectious Disease and Biosecurity, Department of Infectious Diseases, Huashan Hospital and State Key Laboratory of Genetic Engineering, School of Life Sciences, Fudan University, Shanghai, China; b Shanghai Engineering Research Center of Industrial Microorganisms, Shanghai, China; c Department of Laboratory Medicine, Renji Hospital, Shanghai Jiao Tong University School of Medicine, Shanghai, China; d Department of Clinical Laboratory, Shanghai Pulmonary Hospital, Tongji University School of Medicine, Shanghai, China; e Shanghai Key Laboratory of Tuberculosis, Shanghai Pulmonary Hospital, Tongji University School of Medicine, Shanghai, People’s Republic of China; University of Georgia

**Keywords:** diploidy, ploidy change, adaptation, *Candida glabrata*, clinical isolates, haploidy

## Abstract

The human fungal pathogen Candida glabrata is phylogenetically closely related to Saccharomyces cerevisiae, a model eukaryotic organism. Unlike S. cerevisiae, which has both haploid and diploid forms and a complete sexual cycle, C. glabrata has long been considered a haploid and asexual species. In this study, we analyzed the ploidy states of 500 clinical isolates of C. glabrata from four Chinese hospitals and found that approximately 4% of the isolates were in or able to spontaneously switch to an aneuploid (genomic DNA, 1N-2N), diploid (2N), or hyperdiploid (>2N) form under *in vivo* or *in vitro* conditions. Stable diploid cells were identified in 3% of the isolates (15/500). Of particular interest, one clinical strain existed only in the diploid form. Multilocus sequence typing (MLST) assays revealed two major genetic clusters (A and B) of C. glabrata isolates. Most of the isolates (70%) from China belonged to the A cluster, whereas most of the isolates from other countries (such as Iran, Japan, United States, and European countries) belonged to the B cluster. Further investigation indicated that C. glabrata cells of different ploidy forms differed in a number of respects, including morphologies, antifungal susceptibility, virulence, and global gene expression profiles. Additionally, C. glabrata could undergo spontaneous switching between the diploid and haploid forms under both *in vitro* and *in vivo* conditions. Given the absence of an apparent sexual phase, one would expect that the ploidy shifts could function as an alternative strategy that promotes genetic diversity and benefits the ability of the fungus to rapidly adapt to the changing environment.

**IMPORTANCE** The human fungal pathogen Candida glabrata has long been thought to be a haploid organism. Here, we report the population structure and ploidy states of 500 clinical isolates of C. glabrata from China. To our surprise, we found that the ploidy of a subset of clinical isolates varied dramatically. Some isolates were in or able to switch to an aneuploid, diploid, or hyperdiploid form. C. glabrata cells with different ploidy differed in a number of biological respects, including morphology, antifungal susceptibility, virulence, and global gene expression profile. Given the absence of an apparent sexual phase in this fungus, we propose that ploidy switching could be a strategy for rapid adaptation to environmental changes and could function as an alternative to sexual reproduction.

## INTRODUCTION

Fungal infections have become more common in clinical settings during the past several decades due to the increase in the number of immunocompromised individuals (1, 2). Candida glabrata, a species phylogenetically closely related to Saccharomyces cerevisiae, is the second most important fungal pathogen behind Candida albicans in terms of infections (2, 3). C. glabrata differs from C. albicans in several respects. First, the former often exists in the yeast form and is unable to form true hyphae or undergo heritable phenotypic switching (such as white-opaque and white-gray-opaque transitions [4, 5]). Second, C. glabrata has been thought to contain a haploid genome, whereas C. albicans is an “obligately” diploid fungus. Third, C. glabrata often has higher resistance to antifungal drugs, such as fluconazole. A previous study demonstrated that approximately 20% of C. glabrata isolates developed resistance to fluconazole during therapy (6). Fourth, a parasexual reproduction process in C. albicans has been reported. Despite mating type loci similar to those of S. cerevisiae, the process of sexual reproduction has not been observed in C. glabrata.

Genomic plasticity is an adaptive strategy to hostile environments and is often associated with fungal pathogenesis and antifungal resistance in many pathogenic fungi. It has been reported that the generation of aneuploidy is associated with the rapid evolution of new traits in C. albicans, Cryptococcus neoformans, and S. cerevisiae (7–10). We recently found that the emerging fungal pathogen Candida auris could also undergo ploidy alterations to adapt to environmental changes (11). Changes in ploidy state, including the generation of aneuploidy, have a profound effect on many physiological characteristics, such as cell size, growth rate, genomic stability, and transcriptional output (7–10). The number and size of chromosomes vary dramatically and rearrangements of the genome occur frequently in clinical isolates of C. glabrata (12). These genomic changes have been found to be associated with both virulence and antifungal resistance of the fungus (13, 14). Genomic evidence of recombination indicates that C. glabrata may be able to undergo sexual reproduction or have a cryptic sexual cycle (15, 16). Given the lack of an observed/reported sexual cycle in C. glabrata, this genomic plasticity would be fundamentally important to its adaptation to the ever-changing host environment.

In this study, we sought to analyze the phenotypic and genetic diversity of clinical isolates of C. glabrata. By analyzing 500 clinical strains of C. glabrata isolated from four different Chinese hospitals, we discovered two major genetic clades (A and B). Most isolates from China belonged to clade A. To our surprise, approximately 4% of the isolates existed in or were able to spontaneously switch to an aneuploid (genomic DNA, 1N-2N), diploid (2N), or hyperdiploid (>2N) form. Of them, 15 independent strains could exist in the stable diploid form. To our knowledge, this is the first report of the diploid form of clinical C. glabrata strains. We observed that cells with different ploidy forms exhibited different susceptibilities to antifungals, appearances on CuSO_4_-containing medium, and fungal burdens during infection. However, the direct relationship between these phenotypes and ploidy variations and the underlying genetic basis needs further investigation.

## RESULTS

### Collection of clinical isolates of C. glabrata.

We collected 500 clinical isolates of C. glabrata from four hospitals in China. Of these isolates, 211 were collected from Renji Hospital (Shanghai), 98 from Shanghai Pulmonary Hospital (Shanghai), 150 from the First Hospital of China Medical University (Shenyang), and 41 from Guiyang Hospitals (Guiyang). The clinical information for the associated isolates is summarized in Data Set S1 in the supplemental material. CHROMagar medium was used for initial identification of *Candida* species. C. glabrata isolates were verified by sequencing the internal transcribed spacer (ITS) region. All associated patients were adults (18 to 98 years old), including 295 male (59.0%) and 205 female (41.0%) patients. Among the C. glabrata strains, 281 (56.2%) were isolated from patients more than 70 years old, 154 (30.8%) from those 50 to 70 years old, and 65 (13.0%) from those less than 50 years old. The original sources of C. glabrata isolates included sputum (*n* = 263, 52.6%), urine (*n* = 92, 18.4%), genital secretions (*n* = 39, 7.8%), blood (*n* = 37, 7.4%), feces (28, 5.6%), and others (*n* = 39, 7.8%). The strains represented all isolates from a certain period from the corresponding hospital (Data Set S1).

10.1128/msphere.00260-22.3 DATA SET S1 Clinical information for C. glabrata isolates. Download Data Set S1, XLSX file, 0.04 MB. Copyright © 2022 Zheng et al. 2022 Zheng et al. https://creativecommons.org/licenses/by/4.0/ This content is distributed under the terms of the Creative Commons Attribution 4.0 International license.

### Discovery of the diploid form of C. glabrata.

C. glabrata cells normally formed purple or light purple colonies on CHROMagar medium (Fig. S1). Some colonies, which were formed by plating the clinical specimen and identified as C. glabrata, exhibited much darker coloration than the rest. The cells of these colonies were notably larger than normal C. glabrata cells (Fig. S1). We then patched these colonies on yeast extract-peptone-dextrose (YPD) medium and examined the genomic DNA content using fluorescence-activated cell sorting (FACS) assays. As shown in Fig. S1, C. glabrata formed distinct colonies with different levels of coloration. The cells of the darker colonies had a diploid genome, whereas the cells of the regular colonies had a haploid genome. Our findings imply that natural isolates of C. glabrata can exist in the diploid form in the human host.

10.1128/msphere.00260-22.1 FIG S1 Colony and cellular morphologies of two clinical samples (RJ1271 and RJ1372, isolated from throat swabs) of C. glabrata containing both haploid and diploid cells. Four colonies of each sample showing distinct morphologies on CHROMagar medium were replated on YPD medium (containing 5 μg/mL of phloxine B, a red dye). The plates were then incubated at 30°C for 4 days. FACS assays were performed to determine the genomic DNA content. Bar, 10 μm (cells). Download FIG S1, TIF file, 1.3 MB. Copyright © 2022 Zheng et al. 2022 Zheng et al. https://creativecommons.org/licenses/by/4.0/ This content is distributed under the terms of the Creative Commons Attribution 4.0 International license.

### Ploidy variation of C. glabrata isolates.

**FIG 1:**
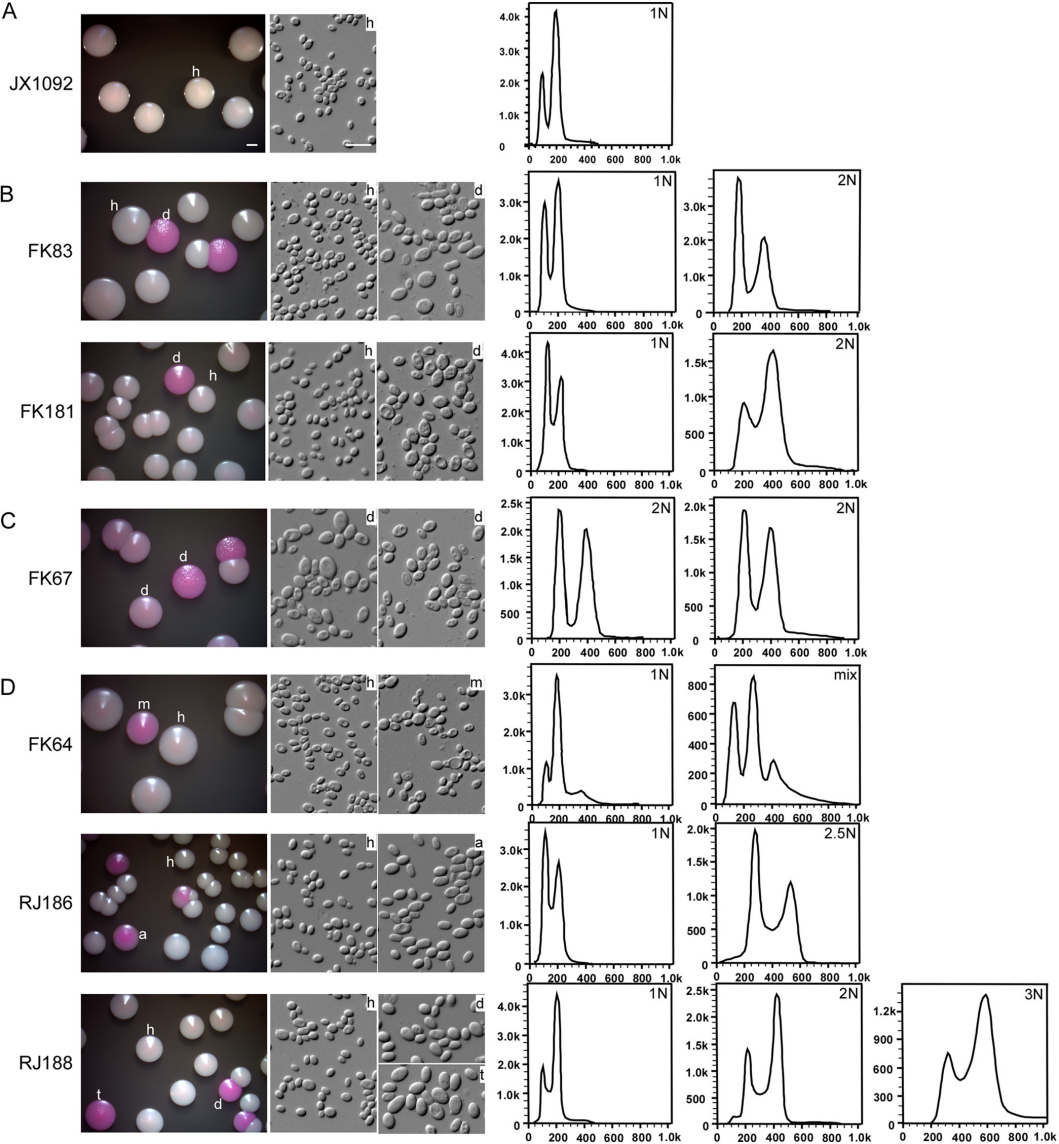
Colony and cellular morphologies of representative isolates of C. glabrata containing haploid, diploid, aneuploid, or hyperdiploid cells. Cells of C. glabrata isolates were initially grown on YPD medium for 3 days. Cells of a representative colony were replated on YPD medium (containing 5 μg/mL of phloxine B) and incubated at 30°C for 6 days. FACS analyses of colonies with different appearances were performed to determine the genomic DNA content. (A) JX1092, a stable haploid isolate; (B) FK83 and FK181, two isolates containing both haploid and diploid cells; (C) FK67, a stable diploid isolate; (D) FK64, RJ186, and RJ188, three isolates containing haploid, aneuploid or hyperdiploid cells. h, haploid; d, diploid; m, mix; a, aneuploid; t, triploid. Bars, 2 mm (colonies) and 10 μm (cells).

We next asked whether ploidy variation was a general feature of clinical isolates. To facilitate the identification of different ploidy forms of C. glabrata, we developed a method by culturing fungal cells on YPD agar containing phloxine B (5 μg/mL) for 6 days at 30°C. As shown in Fig. 1, colonies formed by diploid, aneuploid, or hyperdiploid cells often exhibited a pink or red coloration, whereas colonies formed by haploid cells were shiny and white. Strain JX1092 was a typical haploid strain, the cells of which were relatively small and the colonies white in the presence of phloxine B (Fig. 1A).

Using this method, we analyzed the collected 500 clinical isolates of C. glabrata. Most isolates only formed regular white colonies on YPD medium in the presence of phloxine B. Cells of the colonies were also relatively small. We found that 20 isolates (4.0%) had two or more phenotypes, including white, light pink, and red colonies (Fig. 1B and D). All of the colonies with different phenotypes were verified and identified as C. glabrata by sequencing the ITS region. Microscopic examination demonstrated that the cell size (diameter) of light pink or red colonies (7.5 ± 0.9 μm) was significantly larger than that of white colonies (3.1 ± 0.6 μm). Approximately 20 representative cells were examined for each isolate. FACS analysis confirmed that fungal cells of the 20 isolates with different colorations had distinct genomic DNA content (including diploidy, aneuploidy, and hyperdiploidy). The colony and cellular morphologies and FACS results of several examples with different ploidy forms are presented in Fig. 1. A summary of the ploidy forms of the 500 isolates is presented in Table 1 and Data Set S1. Interestingly, although strain FK67 had two different colony phenotypes, cells of both colony types were diploid (Fig. 1C). This isolate could exist only as the diploid form during infection. However, the coloration levels of both morphologies (pink or red) were higher than that of the haploid form (white). Strain FK67 could represent a special case and reflect the diversity of morphology of C. glabrata. Therefore, a combined assay of the test of genomic content and morphological observation should be performed to accurately determine the ploidy state. Of the 20 samples containing cells with altered ploidy forms (nonhaploid), 15 had diploid cells, 11 had aneuploidy/hyperdiploid cells, and 6 had both diploid cells and aneuploid/hyperdiploid cells. All isolates contained cells of the haploid form, except strain FK67, which was isolated from the respiratory tract and existed only as the diploid form. The proportions of isolates with altered ploidy forms from the respiratory tract, genital tract, body fluid and tissue, and urine/feces were 4.4%, 5.1%, 6.7%, and 2.1%, respectively. The hypergeometric distribution demonstrated that the samples from the respiratory tract had a slightly higher proportion of the diploid form than the samples from other sources (*P* = 0.07). Taken together, these data show that although the proportion was different, perhaps due to the small size of samples, isolates with at least one altered ploidy (>1N) could be found in all four tissue sources (Table 1).

**TABLE 1:** Percentages of clinical samples of C. glabrata containing various ploidy forms^*a*^

Source	No. of samples	% (no.) of samples containing form			
		Haploid	Diploid	Aneuploid or hyperdiploid	Diploid or aneuploid or hyperdiploid
Respiratory tract	272	99.63 (271)	4.04 (11)	1.84 (5)	4.41 (12)
Genital tract	39	100.00 (39)	0 (0)	5.13 (2)	5.13 (2)
Body fluid	45	100.00 (45)	2.22 (1)	6.67 (3)	6.67 (3)
Urine and feces	144	100.00 (144)	2.08 (3)	0.69 (1)	2.08 (3)
All	500	99.80 (499)	3.00 (15)	2.20 (11)	4.00 (20)

a Samples included respiratory tract specimens (sputum, bronchoalveolar lavage fluid, and oral swabs were included), body fluid (abdominal fluid, ascites fluid, blood, pleural fluid, and pus were included), and urine and feces (urine, feces, associated catheters, and drainages were included).

### Genotyping of clinical isolates of C. glabrata.

**FIG 2:**
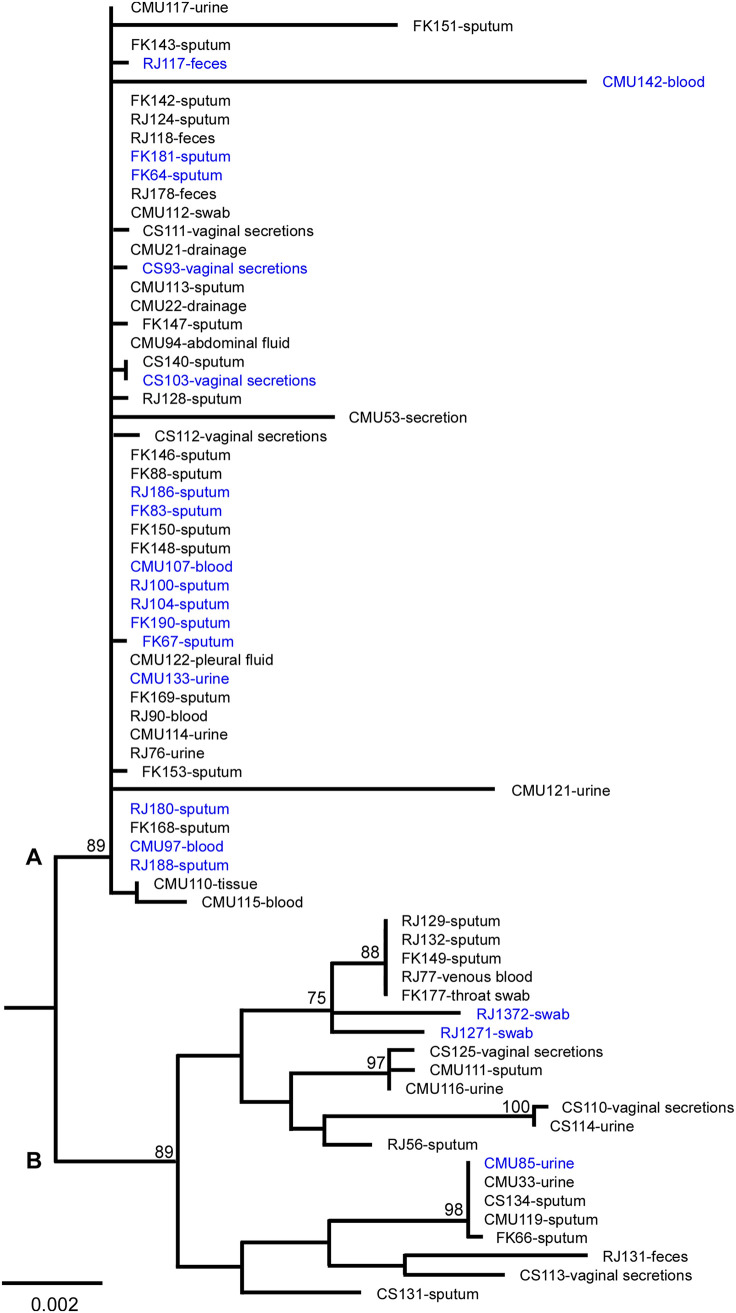
The population structure of 70 C. glabrata isolates from four Chinese hospitals (including 20 with variable ploidy and 50 haploid-only isolates). Multilocus sequence typing (MLST) analysis was performed based on six genes (*FKS*, *LEU2*, *NMT1*, *TRP1*, *UGP1*, and *URA3*). The phylogenetic tree was constructed using the maximum-likelihood (ML) method. The general time-reversible (GTR) model, gamma distribution, and 1,000 bootstraps were adopted. CMU, First Hospital of China Medical University; RJ, Shanghai Renji Hospital; FK, Shanghai Pulmonary Hospital; CS, clinical strains (Guiyang hospitals). According to MLST analysis, C. glabrata strains were divided into two clusters (A and B). The strain names and sources of isolation are presented. The names in blue indicate the clinical strains with diploidy and/or hyperdiploidy forms. Horizontal bars represent expected number of substitutions per site.

We next performed a genotyping analysis using the multilocus sequence typing (MLST) assay. The 20 isolates containing diploid or aneuploid/hyperdiploid cells and 50 haploid-only isolates (randomly selected from the four tissue sources) were examined. Six MLST loci (*URA3*, *UGP1*, *TRP1*, *NMT1*, *LEU2*, and *FKS*) were genotyped according to previous reports (17, 18). As shown in Fig. 2, the 70 isolates were clustered into two major genetic clades (A and B). Most strains belonged to clade A (70%, 49/70), which included 17 (85%, 17/20) samples with an altered ploidy (Fig. 2, in blue). Overall, the percentage of strains with changed ploidy was 2-fold greater in clade A (34.7%, 17/49) than clade B (14.3%, 3/21), implying a bias distribution of samples with an altered ploidy form between the two genetic clades. However, this genetic distribution was not significantly related to the tissue sources or hospitals.

To examine whether there was a difference in genetic distributions between C. glabrata isolates from China and other countries, we retrieved the reported sequences of the six MLST loci of 124 non-Chinese strains from GenBank (www.ncbi.nlm.nih.gov/genbank, accession numbers KX187005 to KX187304, AY771006 to AY771209, and KT763084 to KT763323; strains from Iran, Japan, the United States, and some South American and European countries) (16, 17, 19). The population structure analysis demonstrated that most non-Chinese strains were clustered into clade B (strain names in black, 108/124, Fig. S2), whereas most Chinese isolates belonged to clade A (Fig. S2).

10.1128/msphere.00260-22.2 FIG S2 The population structure of 194 C. glabrata isolates, including 70 isolates from four Chinese hospitals (analyzed in Fig. 2) and 124 isolates from other countries (Iran, United States, Japan, South American countries, and Europe). Multilocus sequence typing (MLST) analysis was performed based on six genes (*FKS*, *LEU2*, *NMT1*, *TRP1*, *UGP1*, and *URA3*). The phylogenetic tree was constructed using the maximum-likelihood (ML) method. The general time-reversible (GTR) model, gamma distribution, and 1,000 bootstraps were adopted. The isolates from Chinese hospitals are highlighted in red. The DNA sequence information for the 124 isolates from Iran, the United States, Japan, South American countries, and Europe was downloaded from the NCBI database (accession numbers KX187005 to KX187304, AY771006 to AY771209, and KT763084 to KT763323). Download FIG S2, TIF file, 0.8 MB. Copyright © 2022 Zheng et al. 2022 Zheng et al. https://creativecommons.org/licenses/by/4.0/ This content is distributed under the terms of the Creative Commons Attribution 4.0 International license.

### Spontaneous ploidy switching in C. glabrata *in vitro* and *in vivo*.

**FIG 3:**
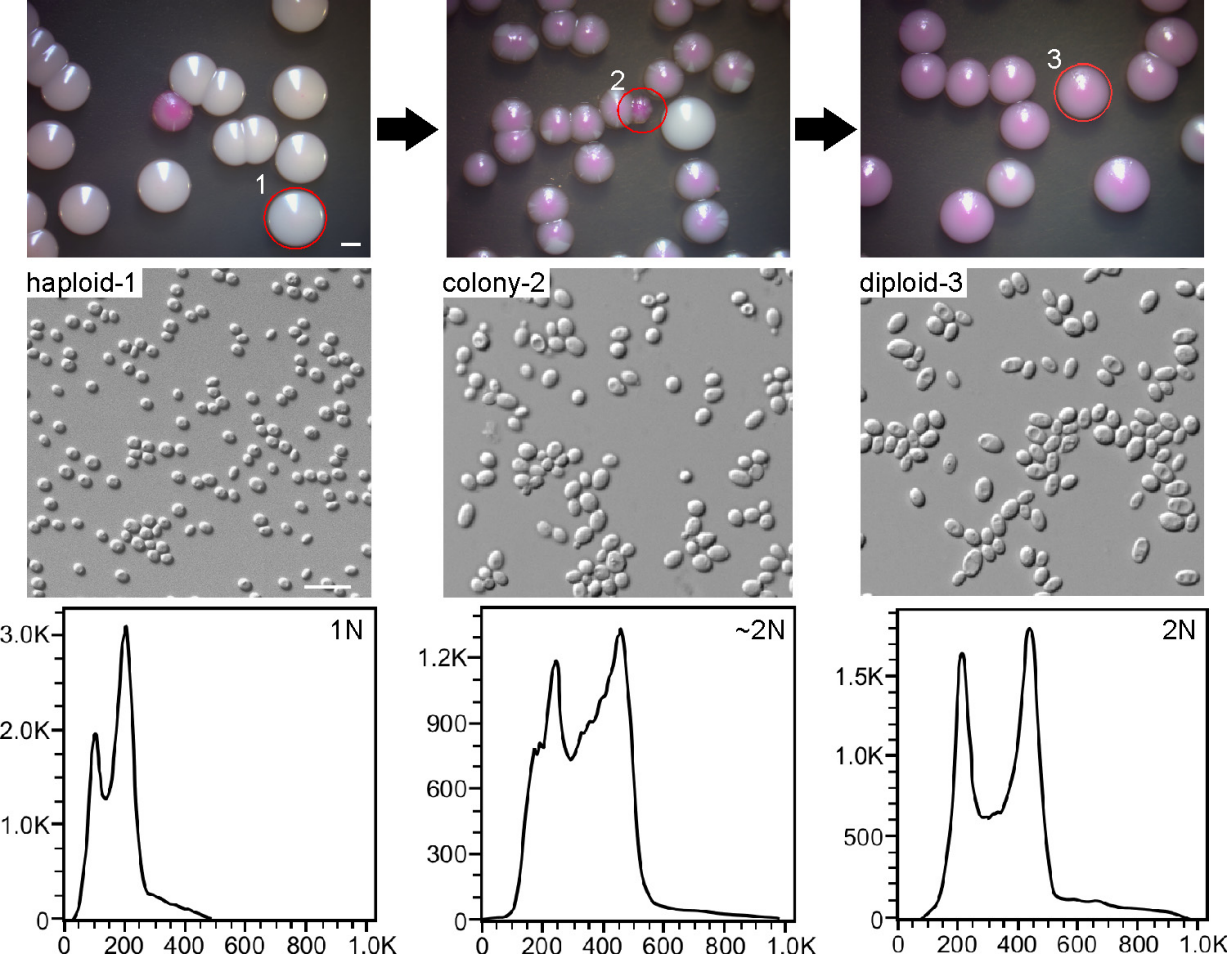
Haploid-diploid switching of C. glabrata on YPD medium. Strain FK83 was used. Haploid cells were initially grown on YPD medium for 3 days. Cells of a homogeneous colony (white) were plated on YPD medium (containing 5 μg/mL of phloxine B) and incubated at 30°C for 6 days. Pink or red colonies were replated on YPD medium, incubated at 30°C for 6 days, and subjected to FACS analysis. When haploid cells were plated, colonies of an intermediate form developed that contained a mixture of both haploid and diploid cells. The frequency of switching from haploid to mixed/diploid form was ~0.6%, whereas the frequency of switching from diploid to haploid was ~1.0%. Bars, 2 mm (colonies) and 10 μm (cells).

Since many samples from the same tissue of a patient could exhibit two or more ploidy forms, we suspected that C. glabrata could undergo spontaneous switching between different ploidy forms under *in vitro* or *in vivo* conditions. To test this hypothesis, we performed switching assays on YPD medium containing phloxine B. Haploid cells of two strains (FK83 and FK181) were initially plated on the medium. After 7 days of growth, approximately 0.3% to 0.6% of colonies appeared pink or red. The pink or red colonies were then replated on YPD medium. FACS analysis demonstrated that these colonies were an intermediate form containing mixed haploid and diploid cells. After another round of plating, homogeneous diploid colonies formed (Fig. 3). To test whether diploid cells of C. glabrata could return to the haploid form, we cultured diploid cells of strains FK83 and FK181 on the same medium plates. Interestingly, diploid cells first switched back to the intermediate form (with a frequency of 1.0% to 1.4%) and then to the haploid form (Fig. 3). These findings demonstrate that only a portion of cells initially switched to the alternative form in the colonies.

**FIG 4:**
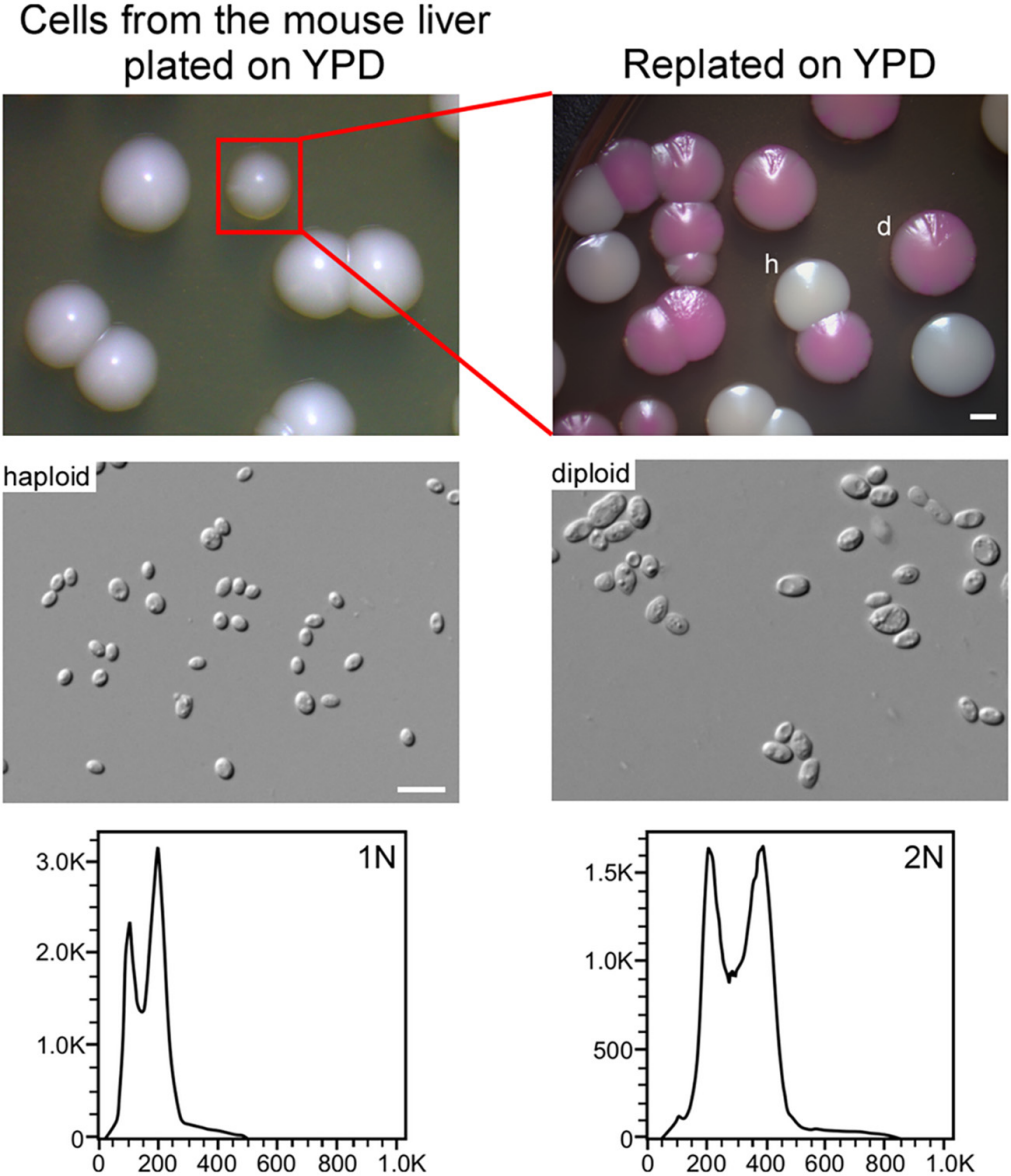
Haploid-diploid switching of C. glabrata in a mouse infection model. Strain FK83 was used. Haploid cells were injected into the mice via the tail vein. Fungal cells were recovered from the liver 24 h postinfection, replated on YPD medium, and incubated at 30°C for 3 days (left). Colonies with darker coloration were replated on YPD medium (containing 5 μg/mL of phloxine B; right) and incubated at 30°C for 6 days. FACS analysis was performed to determine the genomic DNA content. The frequency of switching from haploid to diploid form was ~0.3%, whereas the frequency of switching from diploid to haploid was <0.2%. Bars, 2 mm (colonies) and 10 μm (cells).

To mimic the situation of human infection, we next performed ploidy switching assays using a mouse infection model. The mice were systemically infected with haploid or diploid cells of C. glabrata strains FK83 and FK181 via tail vein injection. After 24 h of infection, the mice were humanely killed, and fungal cells were recovered from the liver tissues and then plated on YPD medium without phloxine B. Some gray colonies (0.1% to 0.3%) were observed and replated on YPD medium containing phloxine B (Fig. 4). After 7 days of growth at 30°C, both diploid (red) and haploid colonies formed in both strains. However, we did not observe diploid-to-haploid conversion in either strain in the systemic mouse infection model (frequency < 0.2%).

Taken together, our data show that C. glabrata is able to undergo ploidy changes under both *in vitro* and *in vivo* conditions, although the switching frequencies are relatively low.

### Cells of different ploidy forms show distinct phenotypes on CuSO_4_-containing medium.

**FIG 5:**
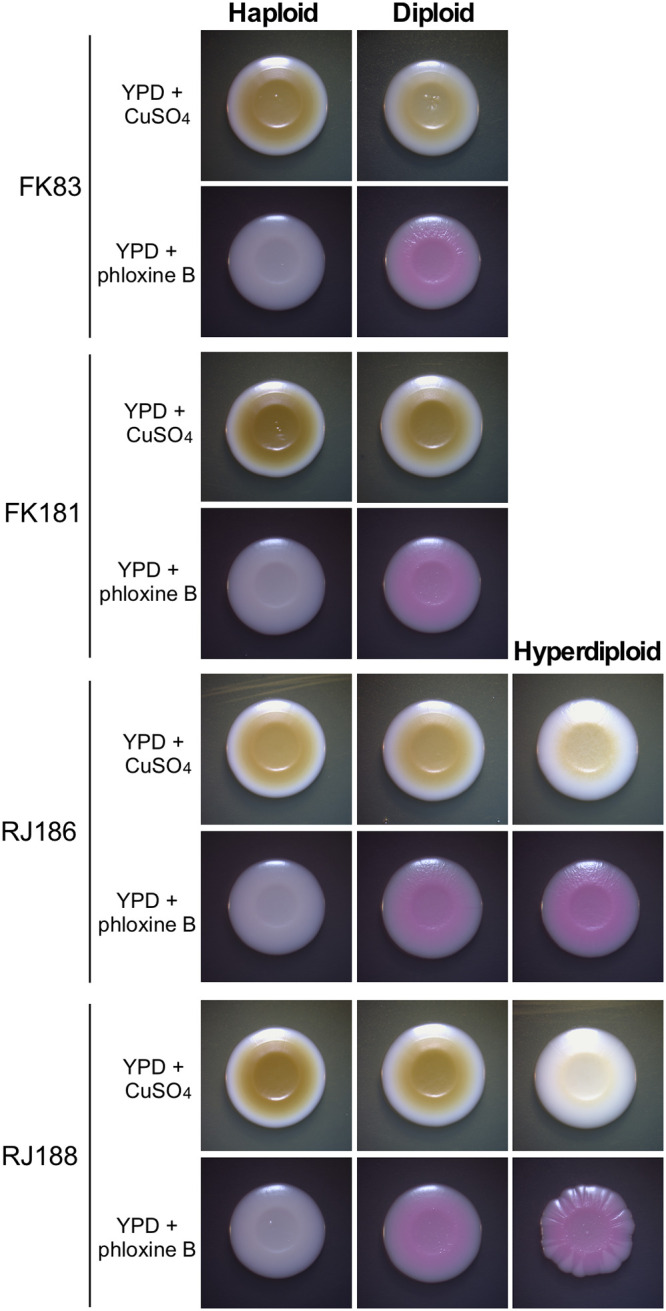
Phenotypes of haploid, diploid, and hyperdiploid cells of C. glabrata grown on YPD+CuSO_4_ and YPD+phloxine B media. C. glabrata cells were initially grown on YPD medium for 3 days. Then, ~1 × 10^5^ cells of each strain in 3 μL ddH_2_O were spotted on YPD+CuSO_4_ (1 mM) or YPD+phloxine B (5 μg/mL) medium and incubated at 30°C for 4 days.

When cultured on YPD+CuSO_4_ medium, haploid C. glabrata strains could form distinct colonies with different colorations due to the distinct ability to convert CuSO_4_ into CuS (20, 21). This ability has been reported to be associated with the virulence of C. glabrata (22). Five representative strains with at least two ploidy forms were spotted on YPD+CuSO_4_ and YPD+phloxine B medium plates and incubated at 30°C for 7 days. As shown in Fig. 5, the order of coloration was hyperdiploid > diploid > haploid on YPD+phloxine B medium, whereas it was the reverse (haploid > diploid > hyperdiploid) on YPD+CuSO_4_ medium. These results imply that C. glabrata cells with distinct ploidy forms can differ in the cellular oxidation-reduction (redox) state.

### Haploid and diploid cells of C. glabrata differ in fungal burdens in a mouse systemic infection model.

**FIG 6:**
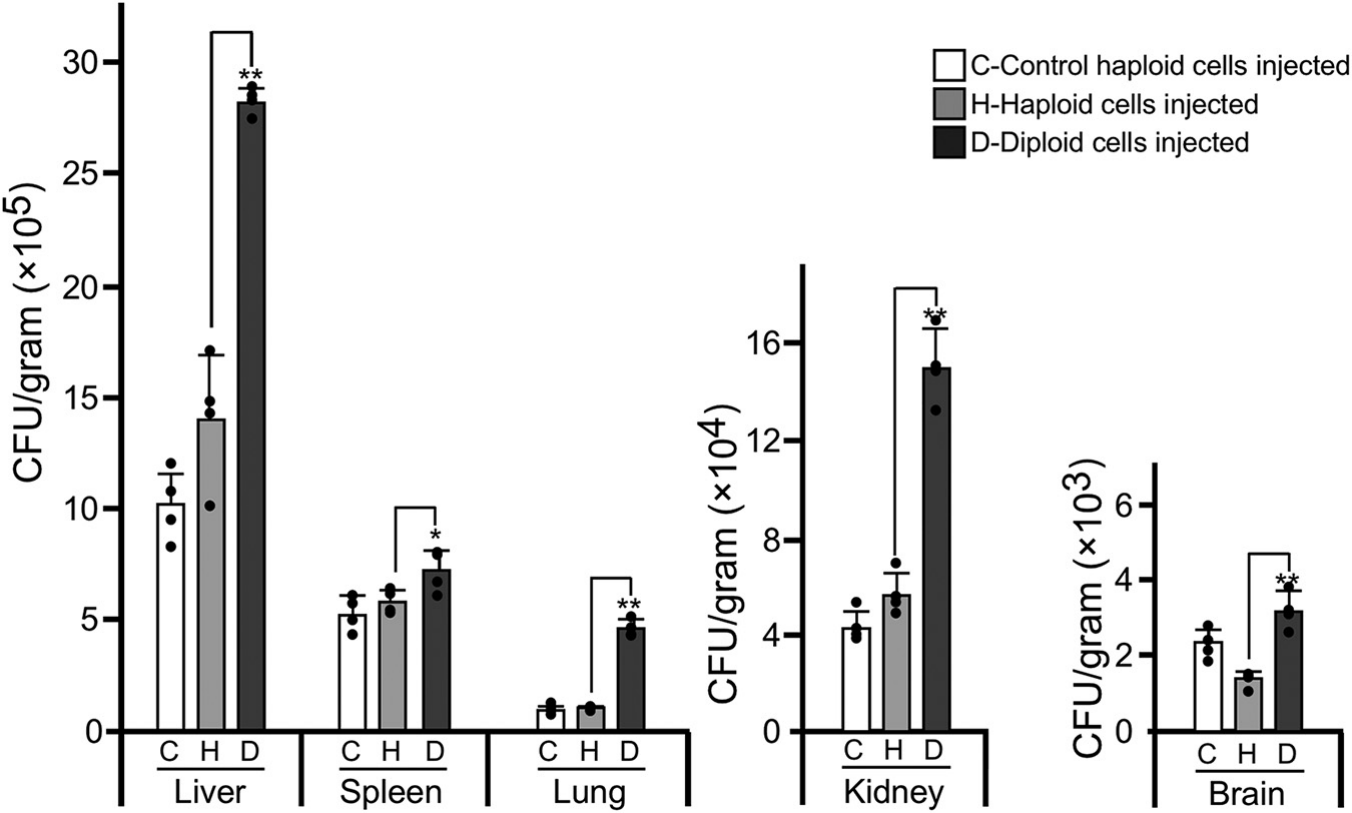
Fungal burdens of haploid and diploid C. glabrata cells in the mouse systemic infection model. Strain used included RJ155 (haploid control), FK83-1 (haploid), and FK83-2 (diploid). Four mice were used for each cell type. Each mouse was injected with 2 × 10^7^ cells of C. glabrata via the tail vein. After 24 h of infection, mice were humanely killed, and fungal burdens of multiple organs were analyzed. The statistical significance of the differences between diploid and haploid values is indicated (*, *P* < 0.05; **, *P* < 0.01; Student's *t* test [two tailed]; the *F* test was used to compare and judge the homogeneity of variance in *t* tests; the standardized effect size for *t* tests was measured by Cohen’s *d*). Black dots represent the CFU data points for each mouse. Error bars represent standard deviations.

Ploidy variations often regulate pathogenesis in fungal pathogens such as C. albicans and C. auris (11, 23). We next tested whether haploid and diploid cells of C. glabrata exhibited distinct levels of fungal burdens using a disseminated candidiasis mouse infection model. Generally, the order of fungal burden in different tissues was diploid > haploid, suggesting that the ploidy level is associated with pathogenesis or the ability of colonization in the host in C. glabrata (Fig. 6). We did not include the hyperdiploid form in this assay, since the genomes of hyperdiploid cells are relatively unstable during infections.

### Haploid, diploid, and hyperdiploid cells of C. glabrata differ in susceptibility to the antifungal itraconazole.

**FIG 7:**
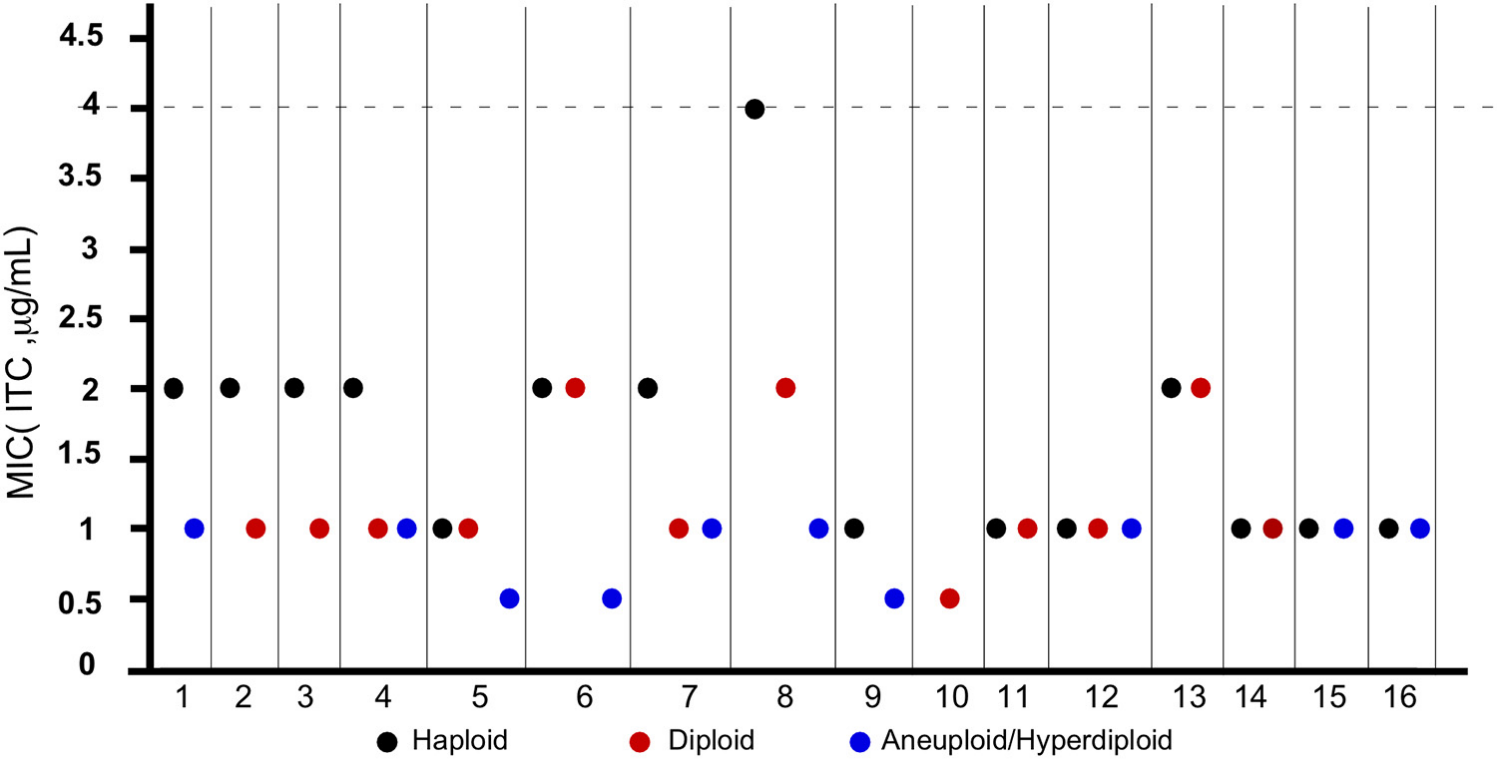
MICs for haploid, diploid, and hyperdiploid C. glabrata cells of itraconazole. MIC assays were performed according to NCCLS document M27-A2 (28). A total of 38 strains derived from Renji Hospital (Shanghai), Shanghai Pulmonary Hospital, and the First Hospital of China Medical University (Shenyang) clinical isolates were tested. The strain information is presented in Data Set S1. Three biological replicates were assessed. The dashed line represents the epidemiological cutoff value of itraconazole (ITC). Candida parapsilosis ATCC 22019 and *Pichia kudriavzevii* ATCC 6258 served as quality control strains.

Itraconazole (ITC) is a first-line antifungal drug. We next tested the susceptibility to ITC in 16 clinical isolates with at least two ploidy forms. As shown in Fig. 7, 56% of the samples (9/16) with different ploidy forms showed a significant difference in terms of MICs. Generally, haploid cells had higher MICs, suggesting that ploidy variations could play a role in the regulation of antifungal resistance.

### Haploid and diploid cells of C. glabrata differ in global transcriptional profiles.

**FIG 8:**
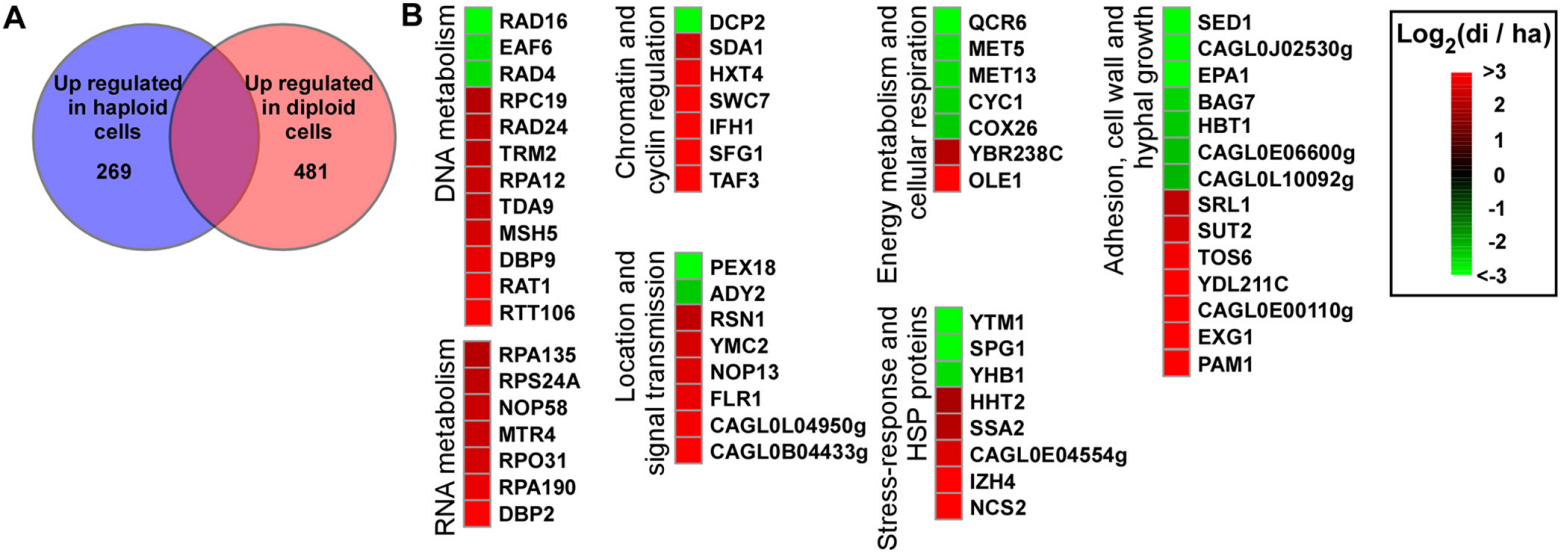
Different gene expression profiles in haploid and diploid cells of C. glabrata. RNA-Seq assays were performed using haploid and diploid cells of strain FK83. (A) Venn diagram of differentially expressed genes. A 2-fold difference cutoff was used to define differentially expressed genes. (B) Representative genes that were differentially expressed. An R package heat map was used to depict selected differentially expressed genes. Log_2_ (di/ha), log_2_ (read counts of diploid cells/read counts of haploid cells). C. glabrata gene names or IDs align with the CGD database (http://www.candidagenome.org/).

We next performed transcriptome sequencing (RNA-Seq) analysis using strain FK83 to elucidate the potential mechanism underlying the biological differences between haploid and diploid cells of C. glabrata. We did not examine the global gene expression profile in aneuploid or hyperdiploid cells because their genomes were not stable. As shown in Fig. 8 and Data Set S2, a total of 750 genes were found to be differentially expressed between the two cell types (2-fold change cutoff, three replicates). Of them, 481 genes were upregulated in diploid cells, and 269 genes were upregulated in haploid cells. These differentially expressed genes are involved in many biological processes, such as DNA/RNA metabolism, chromatin and cyclin regulation, subcellular location, metabolism, and cellular adhesion. Genes associated with chromosome/nucleotide metabolism and the cell cycle were highly expressed in diploid cells (e.g., *TDA9*, *MSH5*, *DBP9*, *RAT1*, and *RTT106* for DNA metabolism and *NOP58*, *MTR4*, *RPO31*, *RPA190*, and *DBP2* for RNA metabolism). This gene expression profile suggested that diploid cells were more active in general metabolism than haploid cells, which was consistent with the higher virulence of diploid cells during infection. Another possibility is that this gene expression difference could be due to the different growth phases of haploid and diploid cells. We observed a similar effect in haploid and diploid cells of C. auris (11).

10.1128/msphere.00260-22.4 DATA SET S2 RNA-Seq data set. Download Data Set S2, XLSX file, 1.0 MB. Copyright © 2022 Zheng et al. 2022 Zheng et al. https://creativecommons.org/licenses/by/4.0/ This content is distributed under the terms of the Creative Commons Attribution 4.0 International license.

Genes involved in energy metabolism and cellular respiration (e.g., *QCR6*, *MET5*, *MET13*, *CYC1*, and *COX26*) were upregulated in haploid cells. Moreover, some genes associated with the stress response, adhesion, cell wall, and morphological transitions were also differentially expressed, suggesting that haploid and diploid cells differ in many biological respects. For example, the haploid-enriched gene *EPA1* encodes an important adhesin that is required for adherence to host epithelial cells, biofilm formation, and antifungal resistance (24). Our findings indicate that haploid and diploid cells of C. glabrata exhibit unique global transcriptional profiles, which could contribute to their distinct responses to host and environmental conditions.

## DISCUSSION

Genomic plasticity is a mechanism of rapid adaptation of human-pathogenic fungi to the hostile environment. For example, aneuploidy and polyploidy are quite common in C. albicans and C. neoformans (8). The model organism S. cerevisiae is able to undergo ploidy changes through both sexual reproduction and spontaneous shift under certain stressful conditions (10, 25). The switch between low and high ploidy forms promotes the generation of *de novo* mutations and diversity of phenotypes (9, 19). In this study, we report the discovery of ploidy variation and haploid-diploid switching in clinical isolates of C. glabrata. Haploid and diploid cells were also able to undergo spontaneous switching under both *in vitro* and *in vivo* conditions (Fig. 3 and 4). More importantly, we isolated diploid cells of C. glabrata from original clinical samples, implying that ploidy variation could occur during host infections. The ploidy variation could be a general feature of clinical isolates. MLST analysis revealed two major genetic clusters of clinical C. glabrata isolates. Most Chinese strains, especially the strains with an altered ploidy form (diploidy or aneuploidy/hyperdiploid), belonged to clade A, whereas most C. glabrata strains from the United States and Iran belonged to clade B. The factors responsible for this genetic variation between different countries remain to be investigated.

Haploid, diploid, and hyperdiploid cells of C. glabrata differed in several respects, including phenotype, antifungal resistance, and global gene expression profiles. On YPD+CuSO_4_ medium, haploid cells formed darker colonies than diploid and hyperdiploid cells (Fig. 5). These chromogenic differences could reflect different redox states and abilities to respond to stresses. Consistently, transcriptional profile analysis indicated that many genes involved in stress response were differentially expressed in C. glabrata haploid and diploid cells. Some isolates with different ploidy levels also differed in susceptibility to the antifungal ITC, suggesting that this change or underlying genetic variations could benefit C. glabrata survival under hostile conditions. In the disseminated mouse infection model, diploid cells of C. glabrata exhibited a higher fungal burden than haploid cells (Fig. 6). Similar results have been reported with C. auris and C. albicans, the diploid cells of which are also more virulent than the haploid cells (11, 23). Consistently, we did not observe diploid-to-haploid switching in the mouse infection model, perhaps due to the increased *in vivo* colonization ability of diploid cells of C. glabrata. These biological differences could benefit fungal cells with distinct ploidy forms to better adapt to different ecological niches during infection or colonization of the host.

Taken together, the data obtained here show that ploidy variation could not only promote the rapid adaptation of C. glabrata to the changing environment but also benefit the evolution of new traits in the long term. The genome of clinical isolates of C. glabrata is extremely unstable (13), which might be due to the frequent change in ploidy forms. The increase in ploidy would result in the generation of aneuploidy and novel mutations. The spontaneous mutation rate of diploid cells has been reported to be much higher than that of haploid cells of S. cerevisiae (21). Moreover, C. glabrata has long been considered an “asexual” fungus. Ploidy switching among the haploid and diploid forms could function as an alternative biological process of sexual or parasexual reproduction. Our findings concerning the diploid form and ploidy variation in C. glabrata would shed new light on its biology and pathogenesis.

## MATERIALS AND METHODS

### Strains and culture conditions.

Clinical isolates of C. glabrata were collected from several Chinese hospitals in Shanghai, Shenyang, and Guiyang in China. Detailed information on these isolates (original tissue sources, ploidy, hospital information, and information on associated patients) is presented in Data Set S1. All samples were plated on CHROMagar medium for initial identification of *Candida* species. The potential C. glabrata isolates were further verified by sequencing the ITS (internal transcribed spacer) region (primers used for PCR were ITS1 [GTCGTAACAAGGTTTCCGTAGGTG] and NL4 [GGTCCGTGTTTCAAGACGG]). YPD medium was used for regular growth of fungal cells. Phloxine B (5 μg/mL), which stains certain types of colonies red or pink, was added to YPD medium. YPD+CuSO_4_ medium (1 mM CuSO_4_) was used for phenotypic analysis of haploid, diploid, and hyperdiploid cells of C. glabrata.

### FACS analysis.

The content of genomic DNA of different isolates was analyzed using FACS analysis according to our previous description (19). C. glabrata cells were initially grown on YPD medium for 72 h at 30°C. The cells were then inoculated and incubated in liquid YPD medium with shaking for overnight growth (at 30°C and 220 rpm). They were then reinoculated into fresh medium (initial optical density at 600 nm [OD_600_] = 0.3), cultured to logarithmic phase (OD_600_ = 1.8), and harvested for flow cytometry analysis. Briefly, fungal cells were washed with double-distilled water (ddH_2_O), resuspended in 300 μL 1× TE buffer (1.2114 g/L Tris, 0.29224 g/L EDTA, adjusted to pH 8.0) and mixed with 700 μL 100% ethanol in 1.5-mL RNase-free tubes. The samples were placed on a vortex mixer (Vortex-Genie 2) for at least 2 h at ambient temperature. The fungal cells were then washed, resuspended with 1× TE buffer, and treated with RNase A (0.3 mg/mL) overnight at 37°C. The cell samples were further treated with proteinase K (0.03 mg/mL) and then incubated for 2 h at 50°C. Fungal cells were collected, washed twice with 1× TE buffer, and suspended in 1× phosphate-buffered saline (PBS). Cells were stained with propidium iodide (25 μg/mL) and used for analysis of genomic DNA content. For each sample, at least 30,000 cells were detected on a FACSCalibur instrument. The software FlowJo 10.4 was used for analyzing the data.

### MLST analysis.

Six gene loci were used for MLST analysis. The primers used were as follows: FKS (5′-GTCAAATGCCACAACAACAACCT and 3′-AGCACTTCAGCAGCGTCTTCAG), LEU2 (5′-TTTCTTGTATCCTCCCATTGTTCA and 3′-ATAGGTAAAGGTGGGTTGTGTTGC), NMT1 (5′-GCCGGTGTGGTGTTGCCTGCTC and 3′-CGTTACTGCGGTGCTCGGTGTCG), TRP1 (5′-AGCACACAGGGATTGTTGTA and 3′-GACCAGTCCAGCTTTTCAC), UGP1 (5′-TTTCAACACCGACAAGGACACAGA and 3′-TCGGACTTCACTAGCAGCAAATCA), and URA3 (5′-AGCGAATTGTTGAAGTTGGTTGA and 3′-AATTCGGTTGTAAGATGATGTTGC). The primers were designed based on previous studies (17, 18). We sequenced the six genes of 70 C. glabrata strains (including 20 isolates with ploidy variation and 50 haploid strains). The 50 haploid strains were randomly selected from the four main sources listed in Table 1 (26 from the respiratory tract, 10 from urine and feces, 8 from body fluid, and 6 from the genital tract). The sequences of 124 C. glabrata strains from Iran and the United States were retrieved from the NCBI GenBank database. Sequences were aligned using MAFFT v7.015b (26). Six alignments were concatenated into a single alignment. The maximum-likelihood (ML) phylogenetic tree was generated using the program RAxML v7.3.2 (27) and the GTRGAMMA model with 1,000 bootstraps.

### MIC assay.

MICs of C. glabrata strains were determined according to the NCCLS document M27-A2 (28) and a previous investigation (29). Fungal cells were initially grown on YPD medium at 30°C for 2 days and then collected and washed with ddH_2_O. Approximately 500 cells were incubated in 200 μL liquid RPMI 1640 medium (1.04% [wt/vol] RPMI 1640, 3.45% [wt/vol] MOPs, adjusted to pH 7.0) using a 96-well U-bottom microplate. A series of concentrations of itraconazole (0.0625, 0.125, 0.25, 0.5, 1, 2, 4, and 8 μg/mL) was tested. Three biological repeats were performed for each isolate. Candida parapsilosis ATCC 22019 and Pichia kudriavzevii ATCC 6258 served as quality controls. The microplates were incubated at 35°C for 24 h. The growth status of cells at different concentrations was determined using a microplate reader. The MIC was determined when the growth state and turbidity of the tested wells were more than 50% lower than those of the control.

### Animal experiments.

All animal experiments were approved by the Animal Care and Use Committee of Fudan University. Four mice (4-week-old female BALB/c) were used for systemic infection for each strain of C. glabrata. Four mice were housed in a cage with an adequate supply of food and water. All the mice were kept in the animal facility for a week of acclimation before being used for infection. C. glabrata strains used included RJ155 (haploid control), FK83-1 (haploid), and FK83-2 (diploid). FK83-1 and FK83-2 are isogenic isolates. The haploid-only isolate (RJ155) served as the control. Fungal cells were initially grown on YPD medium at 30°C for 2 days and then collected, washed, counted, and diluted with 1× PBS. Each mouse was injected with 2 × 10^7^ fungal cells in 250 μL 1× PBS via the lateral tail vein. After 24 h of infection, the mice were humanely killed. The brain, liver, spleen, lung, and kidney tissues were collected, weighed, ground, and plated on YPD medium for the fungal burden and morphological assays.

The YPD medium was supplemented with ampicillin sodium (final concentration, 100 μg/mL) and kanamycin sulfate (final concentration, 50 μg/mL).

### RNA-Seq analysis.

Haploid and diploid cells of C. glabrata (FK83) were incubated in liquid YPD medium for overnight growth at 30°C and reinoculated into fresh liquid YPD medium with an initial OD_600_ of 0.3. Fungal cells were grown to the logarithmic growth stage and collected for total-RNA extraction. Library preparation and sequencing were by the company Berry Genomics (Beijing, China). Briefly, approximately 3 Gb (three gigabases) of reads were obtained by sequencing each library. Three biological repeats were performed for each strain. The sequence data of C. glabrata CBS138 (GenBank accession no. GCA_000002545.2) were used as the reference. Clean reads were mapped to the reference sequence using the software HiSat2 v2.0.5 with default parameters. Transcriptional expression levels of different samples were estimated with StringTie v1.3.3b with default parameters (30). Differentially expressed genes were analyzed with the DESeq2 R package (31). Differentially expressed genes must satisfy two criteria: (i) fold change of ≥2 and (ii) false discovery rates (FDRs) of ≤0.05.

### Microscopy assays.

The colony phenotypes were observed by using a stereomicroscope (NSZ-810+Dig1600; Shanghai Wentek Photonics Technology Co., Ltd., Shanghai, China), and the cellular morphologies were observed using a Leica DM2500 instrument (Wetzlar, Germany). To measure the cell parameters, fungal cells were photographed under a 40× lens objective, and the cell size was measured (20 cells/strain).

### Statistical methods.

Student's *t* test (two-tailed) and the F test were used for Fig. 6. The F test was used to compare and judge the homogeneity of variance. Variance was considered equal if the result was >0.05. Cohen’s *d* was used to measure the standardized effect size for *t* tests. A *d* value of ≥0.2 and <0.5 indicates a small effect; a value of ≥0.5 and <0.8 indicates a medium effect; and a value of ≥0.8 indicates a large effect.

### Spontaneous ploidy switching assays.

For *in vitro* ploidy switching assay, haploid cells of two strains (FK83 and FK181) were initially grown on YPD medium at 30°C for 2 days. Fungal cells of homogeneous colonies (white) were replated on YPD+phloxine B medium at 30°C for 7 days. Approximately 100 cells were plated on each YPD plate. The cells of colonies appearing pink or red were replated on YPD+phloxine B medium and cultured for 7 days at 30°C. Flow cytometry analysis was performed to verify the ploidy state. The frequency of white-to-pink/red colony switching was regarded as the ploidy switching frequency.

For the *in vivo* ploidy switching assay, haploid cells of two strains (FK83 and FK181) were initially grown on YPD medium at 30°C for 2 days. Fungal cells were collected, washed, and diluted with 1× PBS. Cells were used for animal experiments as described above. Fungal cells were recovered from five organs and plated on YPD medium with ampicillin sodium (final concentration, 100 μg/mL) and kanamycin sulfate (final concentration, 50 μg/mL). The colonies with alternative coloration (gray) were replated and cultured on YPD+phloxine B medium at 30°C for 7 days. Flow cytometry analysis was performed to verify the ploidy state. The frequency of ploidy switching was determined.

### Data availability.

The RNA-Seq data set has been deposited in the NCBI Gene Expression Omnibus (GEO) portal (accession no. GSE173617).
