# Supplementary figures and images for: Ploidy Variation and Spontaneous Haploid-Diploid Switching of Candida glabrata Clinical Isolates
Source: mSphere. 2022 Jun 21;7(4):e00260-22. doi: 10.1128/msphere.00260-22 (PMC9429935; doi:10.1128/msphere.00260-22)

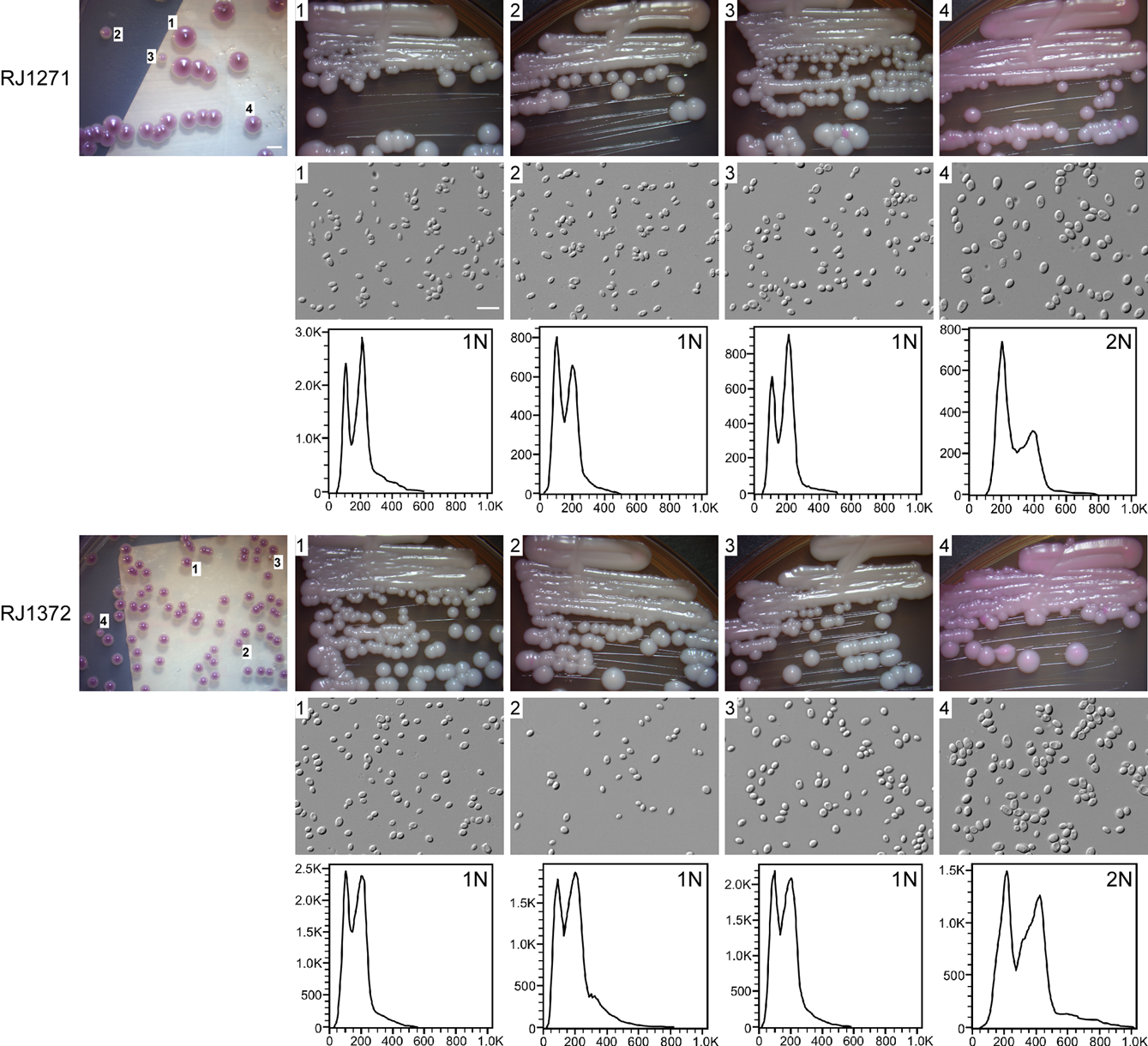

Supplement: FIG S1 [file msphere.00260-22-s0001.tif]

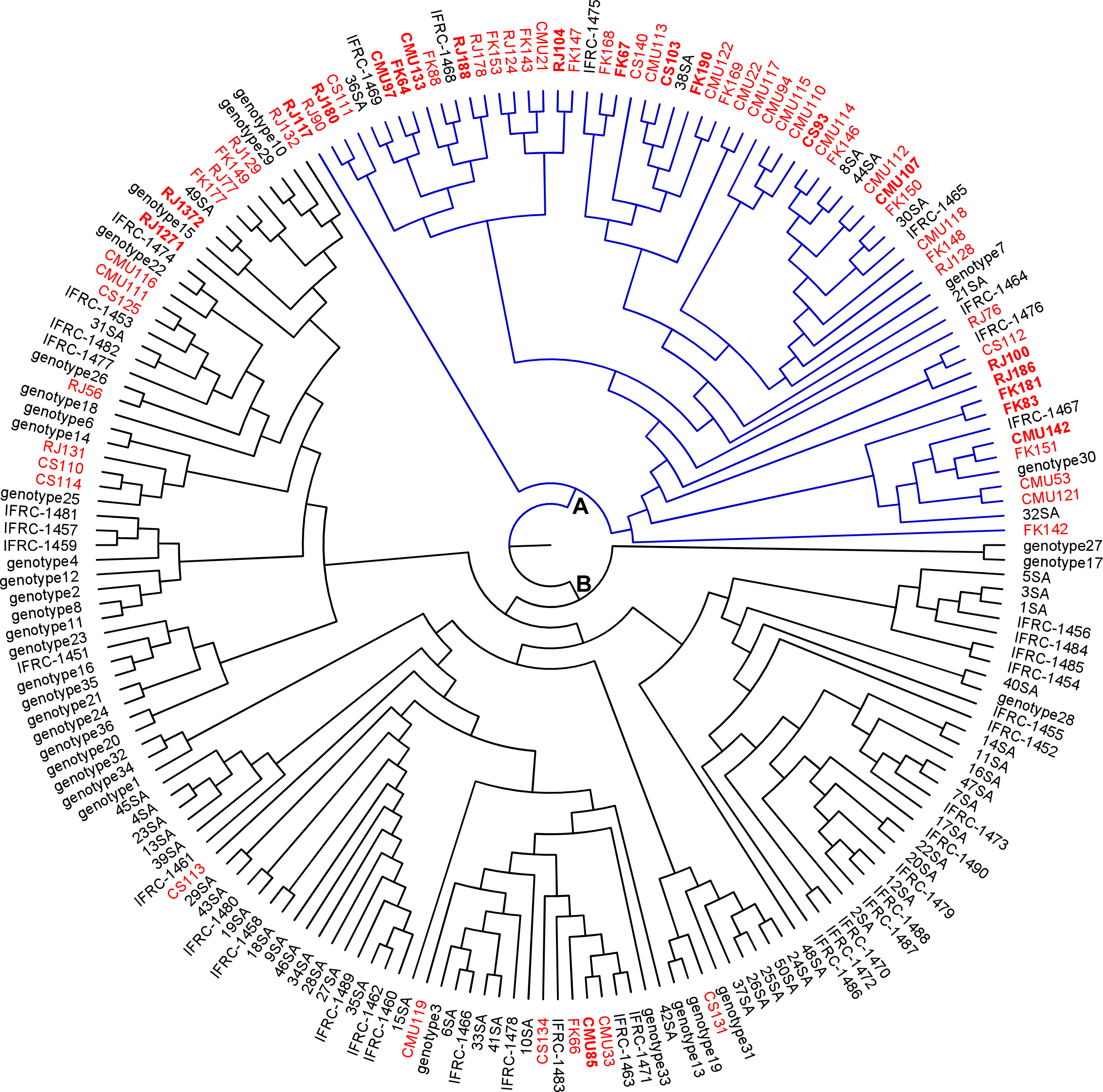

Supplement: FIG S2 [file msphere.00260-22-s0002.tif]
